# The role of hepcidin, GDF15, and mitoferrin-1 in iron metabolism of polycythemia vera and essential thrombocytosis patients

**DOI:** 10.3906/sag-1803-13

**Published:** 2019-02-11

**Authors:** Canan ALBAYRAK, Pınar TARKUN, Elif BİRTAŞ ATEŞOĞLU, Ceyla ERALDEMİR, Özgür Doğa ÖZSOY, Esra TERZİ DEMİRSOY, Özgür MEHTAP, Ayfer GEDÜK, Abdullah HACIHANEFİOĞLU

**Affiliations:** 1 Department of Internal Medicine, School of Medicine, Kocaeli University, Kocaeli Turkey; 2 Department of Hematology, School of Medicine, Kocaeli University, Kocaeli Turkey; 3 Department of Biochemistry, School of Medicine, Kocaeli University, Kocaeli Turkey

**Keywords:** Polycythemia vera, essential thrombocythemia, chronic myeloproliferative neoplasm, GDF15, hepcidin, mitoferrin-1

## Abstract

**Background/aim:**

GDF15, hepcidin and mitoferrin-1 (mfrn-1) are proteins involved in systemic iron regulation. There are no studies in the literature demonstrating the serum mfrn-1 levels in polycythemia vera (PV) and essential thrombocythemia (ET) patients. The aim of this study was to investigate GDF15, hepcidin and mfrn-1 levels in PV and ET patients.

**Materials and methods:**

Ten PV, 17 ET patients, and 27 healthy controls (HCs) were enrolled. GDF15, hepcidin and mfrn-1 values were measured with enzyme-linked immunosorbent assay (ELISA).

**Results:**

GDF15 levels were higher in the myeloproliferative neoplasm (MPN) group (P = 0.002). Hepcidin levels were not different between MPN patients and HCs. The mfrn-1 levels were lower in MPN patients (P = 0.039). Hepcidin, GDF15, and mfrn-1 levels were not different between PV and ET patients. mfrn-1 levels were lower in ET patients than HCs (P = 0.038).

**Conclusion:**

Increased erythropoiesis in MPNs may lead to high GDF15 levels in these patients. However, hepcidin was not suppressed despite the increased GDF15 levels and erythropoiesis in these patients. Decrease in mfrn-1 in MPNs can be the result of its increased turnover due to increased myelopoiesis. It can be hypothesized that similar hepcidin levels in patients and controls and low mfrn-1 levels in patients may be a defense mechanism against erythroid activity and thromboembolic complications.

## 1. Introduction

Myeloproliferative neoplasms (MPN) are clonal hematopoietic stem cell disorders (1). In polycythemia vera (PV) and essential thrombocythemia (ET), there is an increase in all hematopoietic series, but essentially in erythroid and megakaryocytoid series (2). JAK V617F mutation exists in PV, ET, and primary myelofibrosis patients (3). The *JAK2* gene is located on 9p24 (4,5). 

Hepcidin is a small protein primarily released from the liver. It is the main regulator of iron absorption and transportation to the tissues. Plasma iron level and erythropoietic activity regulate the hepcidin level. The increase in the iron level increases the hepcidin production. High hepcidin level reduces dietary iron absorption and the use of iron within the cell; therefore, hepcidin prevents iron accumulation in tissues. High erythropoietic activity suppresses hepcidin. In cases of inflammation and infection, as a host defense hepcidin production increases in order to prevent iron transportation to microorganisms (6).

GDF15 (growth differentiation factor-15) is a member of the TGF-β (transforming growth factor β) family. It is released from mature erythrocytes. It is defined as a hepcidin suppression factor. In disease states where ineffective erythropoiesis is observed (e.g., thalassemia, sideroblastic anemia, congenital dyserythropoietic anemia, etc.), its level increases. In these patients, when GDF15 reaches extremely high levels, it suppresses hepcidin and, therefore, by increasing iron absorption, it contributes to iron overload in the tissues (7).

Iron is an element that is stored and utilized in cellular organelles. Mitochondria use iron in the synthesis of heme molecules (8). Previously, it was not known how the iron molecule was transported to mitochondria. In 2006, Shaw et al. identified a mutated gene in frascati zebrafish with deep anemia and also the protein that this gene synthesizes (3). The protein that is the product of this gene is in the mitochondrial agent transporter family (SLC 25) and identified as mitoferrin (9). Mitoferrin-1 (also known as Slc 25a37) transports the iron necessary for heme synthesis in the developing erythrocytes through the mitochondrial membrane. Mitoferrin-1 in mice is synthesized in high amounts in hematopoietic organs like the fetal liver, bone marrow, and spleen. Mitoferrin-2 is analogous with mitoferrin-1. It is detected in low amounts in all tissues but it is necessary primarily in nonerythroid cells for heme synthesis in hemoproteins and the formation of Fe-sulfide compound (9,10). During erythrocyte maturation, Abcb 10 prolongs the half-life of mitoferrin-1 and therefore increases its concentration. Thus, it enables more iron molecules in the mitochondria for heme and Fe-S compound synthesis (11). Abcb 10 is inhibited by the formed heme molecule and therefore mitoferrin-1 activity reduces. (8). Recent studies have shown that mitoferrin-1 is located as an oligomeric complex together with ferrochelatase and Abcb 10 in mitochondria (11,12).

The purpose of this study is to determine the differences between the iron metabolism of PV and ET patients and healthy controls (HCs) using mitoferrin-1, GDF15 and hepcidin levels since there is an increase in hematopoiesis in MPNs. 

## 2. Materials and methods

In our study, patients who were evaluated in the Department of Hematology and who were diagnosed with PV or ET were included. Patients were diagnosed according to the 2008 WHO criteria (2). All of the enrolled patients were newly diagnosed and patients who received any treatment that could affect iron metabolism, who were previously treated by phlebotomy or hydroxyurea, who had hematologic diseases other than PV or ET, who had active infection or inflammation, and who had decompensated liver, heart, or kidney diseases were excluded from the study. The control group of the study included 27 individuals presenting to the General Internal Medicine Department with no organic diseases. Our study was approved by the local ethics committee. Written consent was obtained from all the participants.

Real-time PCR with a QIAGEN Rotor-Gene Q device was used for the analysis of JAK2 V617F gene mutation. Mitoferrin-1 levels were studied with a Cusabio human mitoferrin-1 (SLC25A37) enzyme-linked immunosorbent assay (ELISA) kit (Cusabio Inc., Wuhan, Hubei, China). Hepcidin levels were studied with a DRG Hepcidin 25 (bioactive) ELISA kit (DRG International Inc., Marburg, Germany). GDF15 levels were studied with a RayBio human GDF15 ELISA kit (RayBiotech Inc., Norcross GA, USA). The results were recorded in ng/mL.

 For statistical analyses, SPSS 20.0 for Windows was used. In comparison of normally distributed variables, the independent sample t-test with two averages or one-way ANOVA analysis was used. The Mann–Whitney U test, Kruskal–Wallis test, and Tukey multiple comparison tests were used in comparison of nonnormally distributed variables. In the comparison of qualitative data, Pearson chi-square and Fisher exact tests were used. P < 0.05 was accepted as statistically significant.

## 3. Results

Twenty-seven patients diagnosed with MPNs [10 PV (37%) and 17 ET (63%) cases] and 27 HCs were included in the study. General characteristics of MPN patients and HCs are shown in Table 1. As seen in Table 1, serum creatinine, uric acid, LDH levels, leukocyte and thrombocyte counts of the patient group were significantly higher than those of HCs. MCV, serum transferrin saturation, iron and ferritin levels were significantly lower than in HCs. 

**Table 1:** Characteristics of patients with MPNs and HC group.

	MPNs, mean ± SD Median (25th–75th percentile)	HCs, mean ± SDMedian (25th–75th percentile)	P
Age (years)	57.07 ± 13.39	56.74 ± 10.97	0.921
Sex (female/male)	11/16	11/16	1.00
Creatinine2(0.6–1.3 mg/dL)3	0.91 ± 0.230.83 (0.72–0.97)	0.77 ± 0.11 (0.76) 0.76 (0.68–0.84)	0.017*
Uric acid2(2.5–7.7 mg/dL)3	5.85 ± 0.995.85 (4.97–6.90 )	4.97 ± 1.345.20 (3.70–6.00)	0.027*
LDH1 (125–220 U/L)3	236.20 ± 55.95	167.25 ± 25.61	<0.001*
Hb2 (12.2–18.1 g/dL)3	14.37 ± 1.9115.10 (12.8–15.6)	14.41 ± 1.23 14.00 (13.5–15.6)	0.736
Hct2 (37.7–53.7%)3	44.27 ± 7.0244.60 (38.8–50.6)	42.98 ± 3.6641.60 (40.7–44.9)	0.324
MCV1(80.0–97.0 fL)3	80.90 ± 10.26	90.86 ± 3.68	<0.001*
Leukocytes1(4.60–10.2 ×103/µL)3	10.75 ± 4.58	6.86 ± 1.04	<0.001*
Platelets1(142–424 × 103/L)3	731.55 ± 252.12	240.92 ± 57.50	<0.001*
CRP 2(0–0.5 mg/dL)3	0.39 ± 0.650.13 (0.07–0.41)	0.28 ± 0.210.25 (0.11–0.42)	0.444
ESR2 (<20 mm/h)3	10.18 ± 12.74.50 (4.00–11.25)	8.18 ± 7.366.00 (2.00–13.00)	0.441
Serum iron1(F: 50–170 µg/dL)3(M: 65–175 µg/dL)3	57.42 ± 34.74	91.18 ± 28.60	<0.001*
Ferritin2(F: 11–306.8 ng/mL)3(M: 23.9–336.2 ng/mL)3	53.45 ± 73.2426.80 (7.30–61.30)	79.59 ± 51.92 63.00 (38.60–99.00)	0.003*
Total iron-binding capacity1(228–428 µg/dL)3	344.69 ± 64.72	318.92 ± 35.31	0.081
Transferrin saturation2 (%)(20–50)3	18.07 ± 12.1717.47 (5.65–27.48)	28.84 ± 9.0727.38 (22.60–32.76)	0.003*

* P < 0.05, statistically significant. 1Independent sample t-test, 2Mann–Whitney U test, 3values given in parentheses are the normal reference ranges. LDH: Lactate dehydrogenase, Hb: hemoglobin, Hct: hematocrit, MCV: mean corpuscular volume, CRP: C reactive protein, ESR: erythrocyte sedimentation rate, F: female, M: male

General characteristics of patients diagnosed with PV and ET in the MPN group are shown in Table 2. In the PV group, the hematocrit levels (P = 0.07) were significantly higher and the thrombocyte counts were significantly lower than in ET patients (P < 0.001) (Table 2). Splenomegaly rate was significantly higher in PV patients (P = 0.003)

**Table 2:** Characteristics of patients with PV and ET.

	PV, mean ± SD Median(25th–75th percentile)(n = 10 patients)	ET, mean ± SD Median(25th–75th percentile)(n = 17 patients)	P
Age (years)	57.80 ± 15.33	56.66 ± 12.59	0.970
Sex (female/male)	4 / 6	7 / 10	1.000
Hb2 (12.2–18.1 g/dL)3	15.34 ± 1.65 15.30 (14.32–16.57)	13.81 ± 1.86 14.30 (11.95–15.40)	0.052
Hct2 (%) (37.7–53.7)3	48.75 ± 5.9650.15 (45.37–51.95)	41.64 ± 6.3543.60 (36.65–45.90)	0.007*
MCV1(80.0–97.0 fL)3	77.99 ± 11.06	82.61 ± 9.69	0.289
Leukocytes1(4.60–10.2 × 103/µL)3	12.18 ± 5.39	9.91 ± 3.97	0.199
Platelets1(142–424 × 103/L)3	567.20 ± 194.31	828.23 ± 235.04	<0.001*
CRP2 (0–0.5 mg/dL)3	0.58 ± 1.010.20 (0.09–0.64)	0.29 ± 0.35 0.11 (0.05–0.46)	0.401
Serum iron1(F: 50–170 µg/dL)3(M: 65–175 µg/dL)3	49.50 ± 42.03	62.37 ± 29.73	0.577
Ferritin2(F: 11–306.8 ng/mL)3(M: 23.9–336.2 ng/mL)3	47.53 ± 79.92	56.92 ± 71.34	0.400
Splenomegaly, n (%)	8 (80 %)	3 (17.6 %)	0.003*
JAK2 V617F mutation positivity, n (%)	10 (100 %)	14 (82.4 %)	0.274
Hemorrhage, n (%)	2 (20.0 %)	1 (5.9 %)	0.535
Thrombosis, n (%)	0 (0 %)	1 (5.9 %)	1.000

*P < 0.05, statistically significant. 1Mann–Whitney U test, 2values given in parentheses are the normal reference ranges. Hb: Hemoglobin, Hct: hematocrit, MCV: mean corpuscular volume, CRP: C reactive protein, F: female, M: male.

When serum hepcidin, GDF15, and mitoferrin-1 levels were compared between the MPN patients and HCs, serum hepcidin levels were similar in the two groups. However, the GDF15 levels of MPN patients were significantly higher than those of the HCs (P = 0.002). In the MPN patients, serum mitoferrin-1 levels were detected to be significantly lower than in the HCs (P = 0.039). An analysis was performed between MPN subgroups and HCs and a subgroup analysis was performed in MPN itself. The data of these analyses are shown in Table 3. No difference was detected between PV and ET patients and HCs in terms of serum hepcidin levels (P > 0.05). However, serum GDF15 levels were significantly higher than those of HCs in both PV and ET patients (P = 0.013, P = 0.034, respectively). The GDF15 levels of PV and ET patients were similar. While the serum mitoferrin-1 levels of ET patients were significantly lower than those of HCs (P = 0.038), no significant difference was observed in other subgroups.

**Table 3:** Serum hepcidin, GDF15, and mitoferrin-1 levels of patients and HCs.

	Hepcidin (ng/mL)Mean ± SD	GDF15 (pg/mL)Mean ± SD	Mitoferrin-1 (pg/mL) Mean ± SD
MPNs (n = 27 patients)HCs (n = 27 patients) P-value	22.50 ± 17.3823.51 ± 12.14 0.806	280.44 ± 138.44178.20 ± 77.030.002*	37.00 ± 10.1942.73 ± 9.630.039*
PV (n = 10 patients)HC (n = 27 patients)P-value	15.31 ± 10.9123.51 ± 12.140.291	301.64 ± 159.84178.20 ± 77.030.013*	40.35 ± 12.9442.73 ± 9.630.791
ET (n =17 patients)HC (n = 27 patients)P-value	26.74 ± 19.3023.51 ± 12.140.756	267.97 ± 127.78178.20 ± 77.030.034*	35.03 ± 7.9542.73 ± 9.630.038*
PV (n = 10 patients) ET (n = 17 patients) P-value	15.31 ± 10.9126.74 ± 19.300.131	301.64 ± 159.84267.97 ± 127.780.734	40.35 ± 12.9435.03 ± 7.950.371

*P < 0.05, statistically significant.

 When hepcidin, GDF15 and mitoferrin-1 levels were analyzed according to the presence of *JAK2* mutation and splenomegaly, it was found that hepcidin levels were significantly lower in the patients with *JAK2* mutation (P = 0.007) (Table 4). There was no difference in terms of hepcidin, GDF15, and mitoferrin levels in patients with and without splenomegaly (Table 4). As the number of patients was low, no analysis was performed according to the thrombosis/hemorrhage history and hepcidin, GDF15, and mitoferrin-1 levels (the number of patients with a history of thrombosis and hemorrhage was 3 and 1, respectively).

**Table 4:** Relationships between hepcidin, GDF15, and mitoferrin-1 levels and presence of JAK2 mutation and splenomegaly.

	Hepcidin (ng/mL)Mean ± SD	GDF15 (pg/mL)Mean ± SD	Mitoferrin-1 (pg/mL)Mean ± SD
JAK2 mutation (+) (n = 24)JAK2 mutation (-) (n = 3)P-value	19.44 ± 13.7847.06 ± 26.950.007*	281.42 ± 143.80272.55 ± 106.130.919	36.51 ± 10.6340.96 ± 4.680.487
Splenomegaly (+) (n = 11)Splenomegaly (-) (n =16)P-value	18.92 ± 20.1224.97 ± 15.420.385	291.30 ± 168.16272.92 ± 119.270.742	39.48 ± 11.6735.29 ± 9.020.303

*P < 0.05, statistically significant.

## 4. Discussion

Hepcidin is a peptide containing 25 amino acids, which was first identified in human urine and plasma (13). There are a few studies in the literature investigating the hepcidin level measurements in MPNs. In one of those studies, four myelofibrosis patients’ urine hepcidin levels were measured and their hepcidin levels were detected to be low (14). In another study by Kwaipsz et al., prohepcidin levels in PV patients were measured and low values were obtained when compared to the control group (15). Especially in patients with iron deficiency, hepcidin levels were lower. It was thought that reduction in hepcidin due to iron deficiency would increase iron absorption and therefore would stimulate erythropoiesis. This would partially explain the disease’s pathogenesis (15). In the literature, there are data indicating that prohepcidin levels are not correlated with urinary and serum hepcidin levels (16). In a study conducted by Tarkun et al., the serum hepcidin levels of the MPN group including PV and ET patients and the HC group were similar (17). Similarly in the present study, hepcidin levels of the MPN group and HC group were similar. Although in PV patients serum hepcidin levels were lower when compared to the HC group, they were not significantly different. In light of this information, it can be suggested that there is a defense mechanism in order to decrease the iron supply and suppress hematopoiesis in MPN patients.

GDF15 production is associated with cellular stress, damage, and apoptosis. In diseases where ineffective erythropoiesis is observed, GDF15 is released from mature erythroblasts in high amounts. High GDF15 levels suppress the hepcidin production. This suppression requires high amounts of GDF15 levels (7,18–20). In the literature, there are several studies indicating the potential role of GDF15 in hepcidin release regulation. Tanno et al. detected that in patients with β-thalassemia where ineffective erythropoiesis was observed, serum GDF15 levels were high and hepcidin levels were low. Again in this study, in human hepatocyte cultures, serum GDF15 and recombinant GDF15 in patients with thalassemia were shown to suppress hepcidin production. High levels (>5000 pg/mL) of GDF15 were required for this suppression (18). In another study that Mast et al. conducted, in high-intensity blood donors where effective hematopoiesis is expected, hepcidin levels were low; however, there was no significant increase in GDF15 levels (21). In the study by Tarkun et al., in the MPN group, serum GDF15 levels were detected to be significantly high; however, no difference was detected in serum hepcidin levels of MPN patients when compared to the control group. In addition, no correlation was observed between GDF15 and hepcidin levels. These results were linked to the fact that GDF15 was not high enough to suppress hepcidin. Moreover, despite the increase in erythropoietic activity and GDF15 levels, it was hypothesized that the normal levels of hepcidin measured in MPN patients could be a defense mechanism to reduce iron utilization in order to prevent erythropoietic activity from increasing (17). In our study, there was no correlation between serum GDF15 and hepcidin levels. 

In our study, in the MPN patients, serum mitoferrin-1 levels were found to be significantly lower when compared to the HC group. Similar to the MPN group, in ET patients, significantly low serum mitoferrin-1 levels were obtained. In the PV patients, although the serum mitoferrin-1 levels were low, there was no statistical difference. In the comparison of PV and ET patients, no statistical difference was observed in terms of mitoferrin-1 values. A few theories can be speculated for low serum mitoferrin-1 levels in MPN patients. First of all, the serum levels may be low because mitoferrin-1 helps iron molecules enter mitochondria and it is used in order to provide iron entry into the mitochondria. In this case, the decrease in serum mitoferrin-1 level can be an indirect indicator of the increase of hematopoiesis in MPN cases. Another reason for low mitoferrin-1 in MPN patients could be a defense mechanism just as there is no decrease in serum hepcidin levels. We believe that low levels of serum mitoferrin-1 may reduce mitochondrial iron uptake and control the myeloproliferation. For this to be conclusive, there is a need for studies investigating the mitoferrin-1 gene expression and synthesis at the cellular level. This cellular defense mechanism can occur through additional undetected factors that inhibit mitoferrin-1 expression. A third mechanism can occur through Abcb 10 forming a complex with mitoferrin-1, ferrochelatase proteins, or a member of this yet-to-be-discovered complex inhibiting mitoferrin directly. All these theories suggest a defense mechanism in order to prevent thromboembolic events, which are a part of the clinical picture and occur as a result of increased hyperviscosity in MPN cases. In PV patients where erythropoiesis is dominant, low serum mitoferrin-1 values were not obtained and this is linked to the insufficient number of patients. In the literature, there is no study about serum mitoferrin-1 measurements in MPN patients. As our study is the first study showing serum mitoferrin-1 levels in these patients, a data comparison could not be carried out.

Hepcidin binds and inhibits ferroportin and stops iron transport from macrophages and iron absorption from intestines (22). When hepcidin is bound to ferroportin, ferroportin phosphorylation occurs (23). Domenico et al. showed that JAK2 is necessary for this phosphorylation; and JAK2’s binding to ferroportin is hepcidin-dependent (24). In the following years, Ross et al. showed that for hepcidin-mediated ferroportin internalization, JAK2 is not necessary and the JAK-STAT pathway is not included in ferroportin phosphorylation (25). In the study conducted by Tarkun et al., hepcidin levels of MPN patients were similar in patients with or without *JAK2* mutation. In the same study, GDF15 levels were significantly higher in patients with JAK2 V617F. Despite these findings, no negative correlation could be shown between hepcidin and GDF15. There is a need for further studies (17). Due to these contradictory literature data, the direct role of *JAK2* mutation on iron metabolism in MPN patients is inconclusive. In our study, within the MPN group, serum GDF15 and mitoferrin-1 values between the patients with and without *JAK2* mutation were similar. We found that in patients with *JAK2* mutation, hepcidin levels were significantly lower, similar to patients with iron deficiency. However, there were only three patients in the *JAK2* negative group, so we think that it is not appropriate to make a conclusion. 

There are several shortcomings of the present study. First of all, the number of patients was low. In addition, we studied the levels of proteins in serum with ELISA and we could not demonstrate the levels of expression of the molecules with genetic methods due to technical insufficiency.

In conclusion, in this study GDF15, hepcidin, and mitoferrin-1 levels of MPN patients were evaluated and this is the only study demonstrating the mitoferrin-1 levels in MPN patients. GDF15 levels in the MPN group were higher than in the HC group, which can be attributed to increased erythropoiesis in these patients. This study revealed that hepcidin was not suppressed despite the increased GDF15 levels and erythropoiesis in these patients. Moreover, mitoferrin-1 levels were lower in MPN patients when compared to HC. It can be hypothesized that similar hepcidin levels in patients and controls and low mitoferrin-1 levels in patients may be a defense mechanism against erythroid activity and thromboembolic complications.

## References

[ref0] (2005). The polycythemias. Hematology: Basic Principles and Practice.

[ref1] (2011). Myeloproliferative neoplasms: molecular pathology, essential clinical understanding, and treatment strategies. J Clin Oncol.

[ref2] (2008). JAK and MPL mutations in myeloid malignancies. Leukemia Lymphoma.

[ref3] (2007). JAK2 mutations and clinical practice in myeloproliferative neoplasms. Cancer J.

[ref4] (2010). Polycythemia vera. Williams Hematology.

[ref5] (2009). The role of hepcidin in iron metabolism. Acta Haematol.

[ref6] (2010). Growth differentiation factor 15 in erythroid health and disease. Curr Opin Hematol.

[ref7] (2009). ABCs of erythroid mitochondrial iron uptake. P Natl Acad Sci USA.

[ref8] (2006). Mitoferrin is essential for erythroid iron assimilation. Nature.

[ref9] (2009). Regulation of mitochondrial iron import through differential turnover of mitoferrin 1 and mitoferrin 2. Mol Cell Biol.

[ref10] (2009). Abcb10 physically interacts with mitoferrin-1 (Slc25a37) to enhance its stability and function in the erythroid mitochondria. P Natl Acad Sci USA.

[ref11] (2010). Ferro chelatase forms an oligomeric complex with mitoferrin-1 and Abcb10 for erythroid heme biosynthesis. Blood.

[ref12] (2001). Hepcidin, a urinary antimicrobial peptide synthesized in the liver. J Biol Chem.

[ref13] (2008). Urinary hepcidin excretion in patients with myelodysplastic syndrome and myelofibrosis. Br J Haematol.

[ref14] (2009). Decreased serum prohepcidin concentration in patients with polycythemia vera. J Zhejiang Univ Sci B.

[ref15] (2009). Hepcidin compared with prohepcidin: an absorbing story. Am J Clin Nutr.

[ref16] (2013). Serum hepcidin and growth differentiation factor-15 (GDF-15) levels in polycythemia vera and essential thrombocythemia. Eur J Haematol.

[ref17] (2007). High levels of GDF15 in thalassemia suppress expression of the iron regulatory protein hepcidin. Nat Med.

[ref18] (2008). Elevated growth differentiation factor 15 expression in patients with congenital dyserythropoietic anemia type I. Blood.

[ref19] (2011). Levels of growth differentiation factor-15 are high and correlate with clinical severity in transfusion independent patients with b thalassemia intermedia. Blood Cells Mol Dis.

[ref20] (2008). genetic analysis of iron metabolism in high-intensity blood donors. Transfusion.

[ref21] (2008). Iron regulation and erythropoiesis. Curr Opin Hematol.

[ref22] (1993). JAK2 associates with the erythropoietin receptor and is tyrosinephosphorylated and activated followings simulation with erythropoietin. Cell.

[ref23] (2009). Hepcidin-induced internalization of ferroportin re- quires binding and cooperative interactions with Jak2. P Natl Acad Sci USA.

[ref24] (2012). Molecular mechanism of hepcidin-mediated ferroportin internalization requires ferroportin lysines, not tyrosines or JAK-STAT. Cell Metab.

